# Physiopathology of nasal and ecnomotopic olfactory receptors: focus on molecular pathways and potential pharmacological modulation

**DOI:** 10.3389/fphar.2026.1905572

**Published:** 2026-09-09

**Authors:** Amedeo Amedei, Cinzia Parolini

**Affiliations:** 1 Department of Clinical and Experimental Medicine, University of Florence, Florence, Italy; 2 Department of Pharmacological and Biomolecular Sciences, Rodolfo Paoletti, Università degli Studi di Milano, Milano, Italy

**Keywords:** ecnomotopic expression, gut microbiome, immunity, metabolic diseases, nasal microbiome, neurodegenerative disorders, olfactory receptors

## Abstract

Detection and discrimination of chemosensory stimuli (usually known as odorants) by the olfactory system exert a crucial role in animal survival. Olfactory receptors (ORs) are a large family of G protein-coupled receptors (GPCRs) mainly expressed by the main olfactory neuroepithelium (OE) at the nasal level and can sense different odorants affecting the electric activity of olfactory sensory neurons (OSNs) eventually generating the sense of smell. Recent evidence suggests that the olfactory mucosa, namely, the OE plus the olfactory mucus, plays a critical role as an immune barrier. Healthy olfactory pathways need intact function and anatomy within the nose itself, the OE, the olfactory bulb, and the central brain structures in charge of olfactory processing. Olfactory loss influences nutrition, safety, and social relationships, and subjects with olfactory impairment have been revealed to be at greater risk for overall mortality. Of note, ORs are not only expressed within the OE but an ecnomotopic expression has also been identified, serving different physiological functions, such as glucose and lipid metabolism and wound healing. It should be noted that ligands of ecnomotopic ORs may originate from naturally occurring or synthetic odor compounds present in the foods and/or food additives, as well as from endogenous compounds produced by host and/or microbiome cells. This review summarizes the anatomy, physiology, and immunology of the human olfactory system. Then, it delves into the ecnomotopic OR expression and signaling pathways. Finally, it reports recent data highlighting how the OR expression can be modulated offering guidance and future directions for clinical translatability.

## 1 Introduction

Animals and humans live surrounded by and immersed in odors. Low-molecular-mass compounds (odorants) convey a myriad of information, and animals have developed a complex olfactory system to detect and transmit them. The ability to interpret the identity and intensity of odors is critical to allow animals to find food, recognize mates, and escape from predators/hazards, thereby ensuring survival, reproduction, and the avoidance of death or life-threatening pathologies. This ability to discriminate a multitude of odors has been performed by the presence in the main olfactory system of many diverse olfactory receptors (ORs) that are able to recognize the odorants, to active signaling pathways, and eventually to translate biochemical stimuli into electrical signals that reach the central nervous system (CNS) *via* topographically defined projections (Smith and Bhatnagar, 2019; Dikeçligil and Gottfried, 2024).

Poor olfaction has been a neglected sensory deficit, but clinical evidence suggests that it may strongly influence an individual’s safety, health, and quality of life (Papazian and Pinto, 2021). This is mostly evident in elderly people where olfactory impairment affects a quarter of the population (Dong et al., 2017). Furthermore, olfactory dysfunction has been suggested as a prodromal symptom for neurodegenerative disorders, such as Alzheimer’s disease (AD), Parkinson’s disease (PD), and dementia, and as a robust predictor for all-cause mortality within 5 years (Quarmley et al., 2017; Chen H. et al., 2017; Cedres and Olofsson, 2024; Wilson et al., 2011; Schubert et al., 2017). Additionally, olfactory impairment has been found in diabetic and obese subjects (Faour et al., 2022).

Although OR expression was initially thought to be localized in the olfactory system, an ecnomotopic expression has been documented and associated with different physiological and pathological conditions. The term “ecnomotopic,” from the Greek meaning beyond the usual place, has recently been adopted replacing the terms “Ectopic” (which usually identifies the presence of an inappropriate pathological site) (Chen et al., 2018) or “Extranasal” (which directly describes the pathological site) (Kalra et al., 2021) to better recognize the physiological functions of these ORs (Di Pizio et al., 2019). Most ligands are found to be naturally occurring or synthetic odorant molecules, including saturated and unsaturated aliphatic acids, alcohols, aldehydes, phenols, terpenes, and gut microbiome (GM) metabolites (Feldmesser et al., 2006; Maßberg and Hatt, 2018; Raka et al., 2022).

Different rodent models with altered olfactory functions have been characterized and linked to behavior and metabolic disorders, and different clinical studies have shown a link between olfaction, behavior, and metabolism (Stark, 2024). Taken together, these studies highlight that ORs may be regarded as translational molecular sensors that bridge chemosensory genomics with tailored diagnosis and therapeutic guidelines.

In addition, transcriptomic and proteomic profiling, as well as new technologies, such as artificial intelligence (AI) and machine learning (ML) frameworks, have improved the knowledge of OR structure and function throughout the body. Most of the studies have been performed in experimental models, but some progress has also been made to resolve OR maps in humans (Dubey et al., 2026).

This review first summarizes the anatomy, physiology, and immunology of the human olfactory system, and then, it provides insights into the ecnomotopic OR expression and its major signaling pathways. Finally, it reports some recent data highlighting how the OR expression can be modulated, offering guidance and future directions for research on ORs and leading to up-to-date clinical guidelines for the management of neurodegenerative and metabolic disorders.

## 2 Search strategy

The search strategy aimed at capturing a broad range of relevant studies focused on molecular and cellular mechanisms to potential therapeutic applications of nasal and ecnomotopic ORs. Indetail, the use of the MeSH tool in PubMed, allowed us to browse through NLM databases. Using this tool, we were able to refine our search and emphasize the relevant studies. The following search terms were combined: “olfactory receptors” AND “human olfactory system” OR “molecular pathways” OR “immunity” OR “microbiome” OR “ecnomotopic expression” OR “neurodegenerative disorders” OR “metabolic diseases.” Only English full articles were selected. The search was updated until May 2026.

## 3 The sense of smell

The human olfactory system is divided into peripheral and central structures. The peripheral structure comprises the main olfactory system, namely, the olfactory neuroepithelium (OE) and olfactory nerves, and the accessory olfactory system, specifically the vomeronasal organ which possesses sensory neurons only during the prenatal period and becomes a vestigial epithelial tube in adults. The central olfactory structures include primary [olfactory bulb (OB), amygdala, piriform cortex, and entorhinal cortex] and secondary (insular cortex and orbitofrontal cortex) central stations for olfactory processing (Smith and Bhatnagar, 2019; Dikeçligil and Gottfried, 2024).

### 3.1 Anatomy of the human olfactory system

**FIGURE 1:**
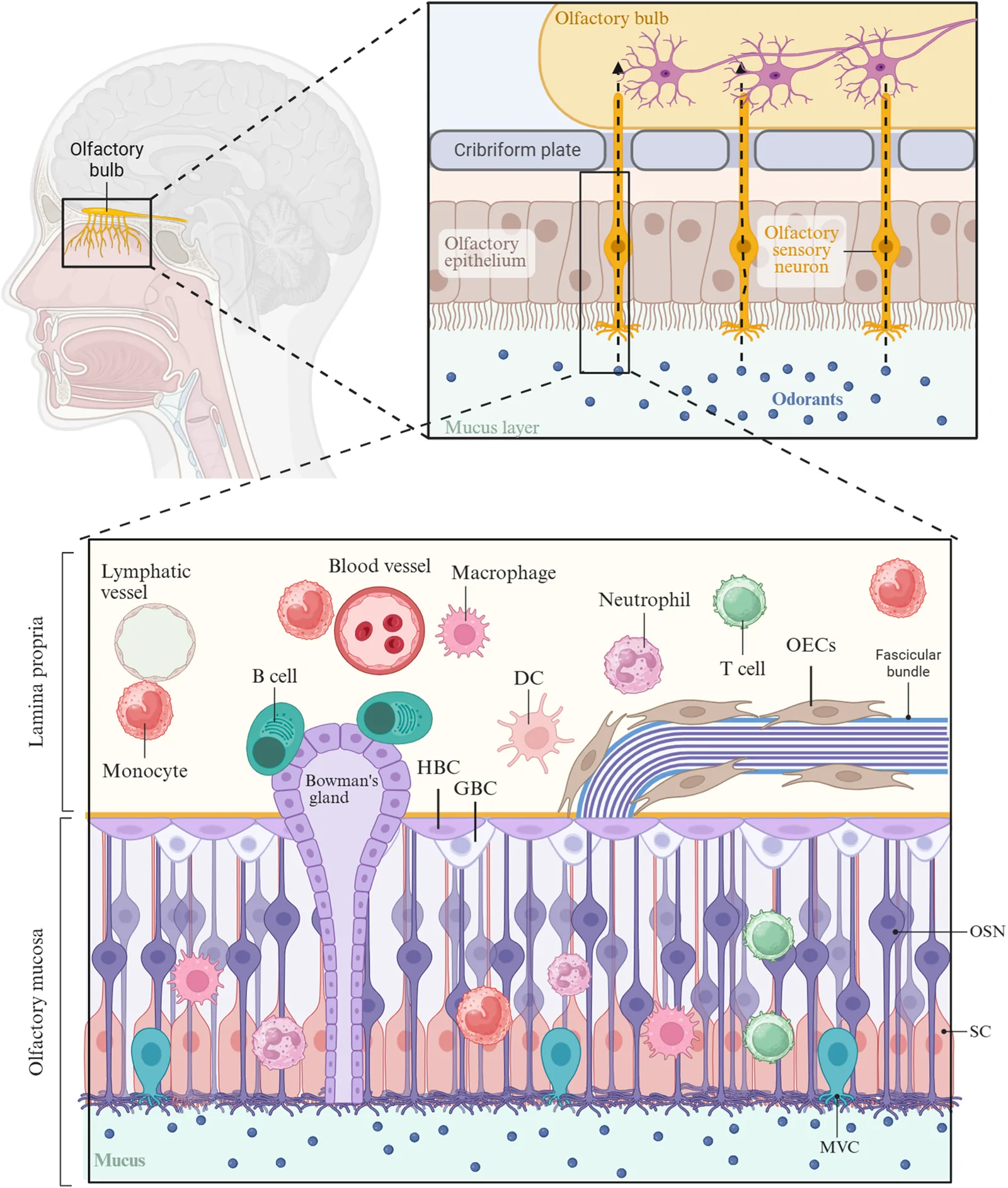
The anatomy of the human olfactory system. Lateral view (up left) of the human nasal cavity and schematic drawing (up right) of the human olfactory system structure. At the bottom of the figure, a schematic drawing of the olfactory mucosa is reported, showing the cell types that compose the olfactory neuroepithelium and the lamina propria. Please refer to the text for the meaning of all abbreviations used in the figure. Figure created with BioRender.com.

Olfactory detection begins in the nasal olfactory structures and proceeds to the first central station for olfactory processing, namely, the OB, which is a brain extension, representing the first sensory structure for direct contact with sensory stimuli and air environmental pathogens (Figure 1) (Rey et al., 2018). The OE, which houses the self-sustaining sensory organ responsible for olfaction, covers the superior and middle turbinates of the nasal cavity (Sharma et al., 2019). Together with the olfactory mucus, this avascular pseudostratified epithelium forms the olfactory mucosa that acts as a defensive barrier against inhaled particles and incoming pathogens (Hewitt and Lloyd, 2021). Indeed, the olfactory mucus is characterized by a combination of mucin 1, mucin 5AC, and mucin 5B proteins (Kennel et al., 2019), having antimicrobial functions, and other proteins, such as olfactory binding proteins (OBPs), having immunomodulatory and antimicrobial properties, as well as facilitating the solubility of odor molecules, which are essential for receptor-mediated olfactory chemosensation (Bianchi et al., 2019; Heydel et al., 2013), whereas the OE houses neuronal and non-neuronal cells. In detail, OE cell types include i) olfactory sensory neurons (OSNs) (Sharma et al., 2019), which house specific odorant receptor proteins, namely, ORs (Amedei and Parolini, 2026); ii) sustentacular cells (SCs) (Liang, 2018), critical for maintaining the structural OE integrity; iii) microvillar cells (MVCs) that express the cystic fibrosis transmembrane conductance regulator (CFTR), a major regulator of mucus secretion and immune responsiveness, which play a relevant role in olfactory tissue homeostasis and submucosal neurogenesis (Hansen and Finger, 2008; Pfister et al., 2015; Genovese and Tizzano, 2018); iv) Bowman’s glands, responsible for the production and secretion of the mucus (Liang, 2018); and v) basal progenitor cells, such as globose basal cells (GBCs) and horizontal basal cells (HBCs), which repopulate the neuroepithelial layer (Figure 1) (Huard et al., 1998). Since these structures are in direct contact with respiratory air containing pathogens and hazardous compounds to maintain sensory function, the OE has a unique capacity to regenerate OSNs. Indeed, OSNs must be replaced by newborn neurons differentiated from basal progenitor cells every few weeks (Liberia et al., 2019). The OSNs are bipolar neurons that project dendrites into the mucosal cavity, wherein their specialized cilia access airway odorants, and axons toward the OB (Sharma et al., 2019). In detail, OSN axons converge in fascicular bundles, forming the olfactory nerve, and tunnel through the olfactory lamina propria first and then the cribriform plate reaching the OB, where they synapse on mitral and tufted cells (Pashkovski et al., 2020). Mitral and tufted cells are altogether named mitral/tufted cells because they are often recognized as functionally homogenous (De Saint Jan et al., 2009). Of note, the fascicular bundles contain immune cells and olfactory ensheathing cells (OECs) (Perera et al., 2020), whereas within the lamina propria, there are lymphatic and blood vessels, whose endothelium allows antigen drainage (Norwood et al., 2019) and forms the blood–olfactory barrier (BOB), respectively. The BOB tight junctions segregate antibodies and other large circulating molecules from the olfactory mucosa (Wellford et al., 2022). Indeed, Wellford et al. (2022) hypothesized that the BOB acting like a functional blood–brain barrier (BBB) extension prevents circulating factors and pathogens from entering the olfactory tissue and reaching the CNS through the “nose–brain” axis. Finally, mitral/tufted cells send their axons through the lateral olfactory tract to higher-order olfactory centers, where they synapse on pyramidal neurons in the olfactory cortex. The olfactory cortex comprises the anterior olfactory nucleus, the olfactory tubercle, the anterior and posterior piriform cortices, the lateral entorhinal cortex, the periamygdaloid cortex, and the anterior cortical nucleus of the amygdala (Perricone et al., 2013). Therefore, unlike other sensory systems, olfactory signals are not integrated in the thalamus before reaching the cortex. However, in these brain regions, olfactory information is integrated with information from other sensory modalities, past experiences, and the animal’s behavioral state to shape olfactory perception, eventually leading to improved discrimination of similar odorants, namely, olfactory learning (Poo and Isaacson, 2009; Li et al., 2008). The piriform cortex seems to play a key role in this complex network producing olfactory perception. Indeed, this area contributes to the discrimination of different odors and to the encoding of the value associated with odor, generating an odor-context memory (Haberly, 2001; Courtiol and Wilson, 2017). Of note, in diverse animals, some odors elicit behavioral responses that seem not to be learned but rather to be innate. Indeed, innate responses appear to be mediated by hard-wired circuits that link specific OSNs to unique neurons in higher brain regions (Marin et al., 2002). Furthermore, the odor perception is not only a simple “bottom-up” pathway but also derived from the combination of this with the “top-down” pathway. In detail, this latter pathway is composed of centrifugal fibers that originate in the olfactory cortex (releasing the excitatory neurotransmitter glutamate) (Balu et al., 2007) and in brain regions containing neurons that release neuromodulators (i.e., norepinephrine, serotonin, or acetylcholine) (Sullivan et al., 1992).

### 3.2 Classification of ORs

Based on their molecular structure, ORs are divided into two subfamilies, class I and class II receptors (Billesbølle et al., 2023; De March et al., 2024). Furthermore, class I ORs are categorized into three subfamilies (such as 51, 52, and 56), whereas class II ORs are categorized into 14 subfamilies, ranging from number 1 to number 14 (Olender et al., 2020). Consequently, the official nomenclature for ORs is as follows: the root “OR” followed by a family number, i.e., OR51 and OR1, a subfamily letter, i.e., OR51E and OR1A, and a number identifying the specific receptor within the subfamily, i.e., OR51E2 and OR1A1 (Olender et al., 2020). This complexity mirrors the extensive adaptability and specificity of ORs in detecting different types of structurally diverse molecules. In addition, genetic polymorphisms within OR genes may strongly influence individual olfactory phenotypes, including olfactory acuity, highlighting the seminal role of genotypes in the perception of odors (Trimmer et al., 2019). Transcriptomic mapping reveals a significant enrichment in abundance for human OSNs that detect key food odorants, whereas a similar enrichment was found for mouse semiochemicals in mouse OSNs (Saraiva et al., 2019). Accordingly, a recent systematic review and meta-analysis revealed that copy number variations (CNVs) in OR genes are associated with obesity risk and that olfactory performance is inversely related to body mass index. In addition, bariatric surgery procedures appear to be linked to an improvement in the olfactory threshold (Matiashova et al., 2023). Furthermore, African populations generally retain a higher number of OR genes than European or Eat Asian cohorts, mirroring distinct evolutionary pressure associated with the diet and environment (Gilad and Lancet, 2003; Niimura, 2009; Phillips et al., 2021). Shadravan (2013) reported sex-biased CNV in the OR gene family, which is markedly influenced by ethnicity. However, in terms of the overall number of OSNs, data from mice suggest that there is no difference between male and female individuals, but no direct comparisons have been performed on humans (Kawagishi et al., 2014). When the OR expression was evaluated, contrasting results have been obtained for mice, ranging from higher levels in male individuals than in female individuals to no differences between the sexes (Shiao et al., 2012; Kanageswaran et al., 2015). Verbeurgt et al. (2014) found no sex differences in the number or expression of human ORs in an expression profiling study on 365 ORs.

Even though recent progress into the OR structures has been made (Billesbølle et al., 2023; De March et al., 2024; Choi et al., 2023) and diverse integrated platforms are available (Sharma et al., 2022; Ollitrault et al., 2024; Lalis et al., 2024), the specific odorants recognized by each OR (only 20% of human ORs are now deorphanized) and the downstream activated pathways are still under investigation, primarily caused by challenges in studying OR structures. These challenges encompass the low OR expression in heterologous cells and the limited solubility of most volatile odorants (Billesbølle et al., 2023; De March et al., 2024; Choi et al., 2023). Regarding the low expression, it has been demonstrated that ORs require palmitoylation (Moench et al., 1994) and olfactory-specific chaperones to be correctly targeted to the surface of cells (Kurtz et al., 2020). In detail, the cytoplasmic tail (i.e., close to the carboxy terminus) of the receptor undergoes palmitoylation (Moench et al., 1994). There is strong evidence that OR palmitoylation may 1) be implicated in the efficient expression of the functional receptor at the cell surface and 2) influence the access to phosphorylation sites in the receptor, thereby inhibiting rapid desensitization (see below). The ability of OSNs to express cloned receptors may depend on the presence of some olfactory-specific chaperone or co-factor needed for functional receptor expression in these cells (Moench et al., 1994). As documented by Kurtz et al. (2020), some of these trafficking proteins have been identified, but some ORs, although produced in transfected cells, were unable to achieve membrane surface expression even in the presence of the known trafficking proteins, suggesting the need for further research. In addition, membrane composition and organization influence the OR downstream pathways. For instance, lipid rafts, highly organized microdomains enriched in phospholipids, glycosphingolipids, and cholesterol, regulate the signal transduction by facilitating the membrane interaction between activated Galpha subunits and ACIII (Oh and Schnitzer, 2001), whereas non-lamellar membrane microdomains influence the translocation/activation of downstream enzymes from the cytosol to membranes (Escriba et al., 1995). Finally, the lack of receptor-specific antibodies increases the complexity of this field. Cell-free protein expression systems may represent an alternative for expressing ORs in heterologous systems.

## 4 The olfactory immunology

Olfactory mucosa is a CNS mucosal barrier and a critical component of pathogen defense, being the sole border between the external environment and higher brain regions. Loss of its homeostasis may create marked health risks, including loss of the sense of smell and, notably, of the integrity of the barrier function against infection. Indeed, microbes within the nasal cavity may take advantage of the olfactory pathway and access the subarachnoid space and the OB, evading both the neurovascular and meningeal barriers, eventually causing life-threatening neurological diseases (Phillips et al., 2013; Schrauwen et al., 2012; Munster et al., 2012). Immune protection of the olfactory mucosa plays a crucial role against respiratory pathogen transmission and neurotropic microbial invasion and is guaranteed by resident adaptive and innate immune cells, such as B and T cells, macrophages, dendritic cells, and neutrophils, which carefully patrol these areas (Buckley and McGavern, 2022; Moseman et al., 2020). Of note, the seminal role played by the immune system for the OE integrity has been derived from different studies performed after the COVID-19 pandemic (Torabi et al., 2020). Immune cells, mainly coming from the nasal-associated lymphoid tissue (NALT) (Tacchi et al., 2014), enter the OE and exert their modulatory activities both under physiological and pathological conditions (Doty and Kamath, 2014; Rustenhoven and Kipnis, 2019). Consistent with these data, a resident population of macrophages regulates neural basal progenitor cell proliferation, differentiation, maturation, and survival of OSNs through the modulation of the expression of different genes (Durante et al., 2020). In addition, Rattazzi et al. (2015) detected an impaired sense of smell because of OE atrophy, OB glomeruli disorganization, and NALT impairment in recombinant-activating gene1 (RAG1) knock-out immunodeficient mice. Following an OE lesion, transient elevations in tumor necrosis factor (TNF) and interleukin (IL)-1beta trigger the increases in vascular blood flow and permeability, promoting the recruitment of innate immune cells, including neutrophils and macrophages, which can neutralize and contain pathogens (Chen M. et al., 2017). During this stage, neuronal death is observed, which is limited by the rapid OE renewal. Immune components may alter mucus secretion and composition [secretion of different cytokines, such as IL-2, IL-5, IL-6, IL-8, IL-10, IL-13, and IL-33, and chemokines, such as monocyte chemoattractant protein-1 (MCP-1) and granulocyte-macrophage colony-stimulating factor (GM-CSF) (Manzini et al., 2015; Schlosser et al., 2016)] and modulate ionic balance and non-neuronal OE cell types, leading to a dramatic expansion of the olfactory submucosa causing axonal damage. Axonal disruption and loss of central connectivity may trigger OSN apoptosis, justifying the observed inflammatory-induced olfactory loss (Bryche et al., 2021). This phenomenon is reversible, as demonstrated by Lane et al. (2010) using a mouse model in which TNF expression is induced in a temporally controlled manner specifically within the OE. However, during chronic inflammation, basal progenitor cells switch from a regenerative phenotype to that participating in immune defense by the activation of the nuclear factor kappa B (NF-κB) pathway, leading to the upregulation of chemokines and cytokines, such as MCP-1 (Chen et al., 2019; Parolini, 2025a). This pathological condition could result in diminished OE regeneration, eventually causing long lasting or permanent anosmia.

In addition, evidence suggests that other cells are crucial for the homeostasis of the olfactory mucosa. Following SC injury, the chemosensory function and the neuroepithelial layer are altered because the olfactory mucosa is compromised. Altered gene expression in SCs also contributes to chronic inflammation, which can result in persistent anosmia (namely, loss of smell). In addition, a recent study demonstrated that SCs contribute to the regulation of the local inflammatory response, easing the recruitment of immune cells to the olfactory mucosa (Dietz et al., 2024). Upon airway allergen exposure, MVCs upregulate the gene expression of IL-25 and Ltc4, thereby promoting olfactory stem cell proliferation, allowing the speculation that these cells may orchestrate the immune response and neurogenesis (Verma et al., 2022; Ualiyeva and Bankova, 2022).

OECs surrounding OSN axon bundles act as a relevant glial component of the olfactory nerve (Beiersdorfer et al., 2020), whereas microglia localized in the OB are crucial for maintaining homeostasis during development and in response to injury and infections (Meller and Greer, 2025). OECs behave in two different ways based on their localization: OECs within the OB drive axon regeneration, whereas olfactory mucosa OECs produce inducible nitric oxide synthase (iNOS) and cytokines to address bacterial invasion and phagocytose-infected or dying olfactory axons (Lan et al., 2022; Harris et al., 2009; Denaro et al., 2022). It has been observed that the phagocytic capacity of OECs is greater than that of olfactory mucosa macrophages and seems to be enhanced by the activation of the MIF/HTRA1 (macrophage migration inhibitory factor/high temperature requirement serine protease A1) pathway (Wright et al., 2020).

Finally, additional BOB characterization and deeper investigation of its role in immune defense are needed to shed new light on the unique immunological milieu of the olfactory mucosa. These advancements could lead to better tailored therapeutic strategies for diseases, such as COVID-19.

## 5 Ecnomotopic ORs

Although ORs are primarily expressed in OSNs, OR genes have also been identified throughout the body in different tissues (Feldmesser et al., 2006; Maßberg and Hatt, 2018; Raka et al., 2022). This ecnomotopic expression essentially derives from mRNA identification through qRT-PCR, microarray analysis, and next-generation sequencing due to the lack of receptor-specific antibodies. The expression levels of these ecnomotopic ORs are lower than those of the same receptors in the OE (Lee et al., 2019). Furthermore, the number of ecnomotopic ORs significantly diverges among the different tissues, and many of these ORs may be co-expressed in multiple tissues, suggesting that they do not respect the “one neuron, one receptor” rule (Lee et al., 2019; Hanchate et al., 2015). Of note, evidence demonstrates that the ecnomotopic expression of some ORs is upregulated in several cancers and diseased tissues, leading to the speculation that these ORs may represent novel biomarkers and therapeutic targets for diagnosis and treatment of human diseases (Ranzani et al., 2017; Neuhaus et al., 2009; Cui et al., 2013; Weber et al., 2018). However, the function of most ecnomotopic ORs remains to be completely established, especially in metabolic tissue, as their ligands and specific mechanism of action are still a matter of investigation (Ren et al., 2024). It should be underlined that ligands of ecnomotopic ORs may originate from naturally occurring or synthetic odor compounds present in foods (such as cilantro, onions, mint, and celery) and/or food additives (such as flavorings), as well as from compounds produced endogenously by human and/or microbiome cells. For instance, asprosin, a fasting-induced gluconeogenic hormone that promotes glucose production in the liver and stimulates appetite in the hypothalamus (Romere et al., 2016; Duerrschmid et al., 2017), has been recently recognized as a ligand of the mouse OR 734 (Olfr734, mouse homolog of OR4M1), which can promote hepatic glucose production during fasting conditions and in obesity (Table 1) (Li et al., 2019). Short-chain fatty acids (SCFAs), mainly acetate, propionate, and butyrate, generated by the GM fermentation of dietary fibers, are the ligands of diverse ORs localized in the gastrointestinal epithelium, renal juxtaglomerular afferent arteriole, vascular smooth muscle cells, and heart (Bellono et al., 2017; Billing et al., 2019; Pluznick et al., 2009; Pluznick et al., 2013; Chang et al., 2015; Jovancevic et al., 2017). Among these receptors, Olfr78/OR51E2 and Olfr558/OR51E1 expression was detected in colonic enteroendocrine cells, in which their activation regulates maturation, gene expression, and serotonin (5-HT)/glucagon-like-peptide-1 (GLP-1) secretion from enterochromaffin cells (Lund et al., 2018; Han et al., 2018; Dinsart et al., 2024), and in vascular smooth muscle cells and carotid body, where they play a role in blood pressure regulation and the cardiovascular–kidney–metabolic axis (Pluznick et al., 2013; Chang et al., 2015; Beito et al., 2024; Shi et al., 2024). These data confirm the local and peripheral effects of SCFAs and strengthen the influence of the GM composition and function in the host homeostasis (Peters et al., 1992; Amedei et al., 2025). Indeed, different studies demonstrated that decreasing the GM biomass with antibiotics or deleting the *Olfr78* expression in a genetically modified mouse model (namely, *Olfr78*-deficient mice) alters the blood pressure control (Pluznick et al., 2013) or causes dysbiosis, increases inflammation, and impairs efficient tissue regeneration (Dinsart et al., 2024; Kotlo et al., 2020), respectively. In agreement with these data, Olfr544 activation by azelaic acid orchestrated the metabolic interplay between the liver and adipose tissue, promoting the release of stored fats from adipose tissue and shifting the fuel preference to fats in the liver and brown adipose tissue, eventually leading to a reduced adiposity (Wu et al., 2017). In addition, Wu et al. (2021) demonstrated that Olfr544 activation by the same ligand induced GLP-1 secretion by enteroendocrine cells while modulating fecal metabolome and GM, resulting in anti-inflammatory effects (Table 1). These data were confirmed by a recent study showing that another Olfr544 ligand, eugenol, was effective in suppressing diet-induced steatohepatitis in mice through two mechanisms: 1) directly, by enhancing hepatic fatty acid beta-oxidation and lipolysis; 2) indirectly, through GM remodeling, characterized by higher abundances of *Lactobacillus johsonii and L. reuteri*, consequently increasing the levels of two hepatoprotective GM metabolites, namely, 3-indolepropionic acid (IPA) and 5-hydroxyindole-3-acetic acid (5-HIAA), which inhibit the hepatic synthesis of lipids (Wang et al., 2025). In addition, these authors detected diminished serum levels of IPA and 5-HIAA in patients with non-alcoholic fatty liver disease (NAFLD), highlighting the clinical translation of their experimental results (Wang et al., 2025).

**TABLE 1:** Physiological functions of enomotopic expressed ORs.

Human olfactory receptor	Mouse ortholog	Tissue	Ligand	Signaling	Study	Biological effect	Disease
OR4M1	Olfr734	Liver	Asprosin	ACIII/PKA/CREB	*In vivo*	↑hepatic glucose production	Diabetes
OR51E2	Olfr78	Colon	SCFAs	ACIII/cAMP/CNG Ca+2 channels	*In vitro In vivo*	↑serotonin and GLP-1 secretion; dysbiosis; ↑intestinal inflammation	Diabetes and inflammatory bowel disease
		Vascular carotid body renal juxtaglomerular apparatus	SCFAs	cAMP	*In vitro In vivo*	↑blood pressure	Hypertension
​	Olfr544	Liver and adipose	Azelaic acid	ACIII/PKA/CREB/HSL	*In vivo*	↑hepatic FAO; ↑lipolysis; ↓adiposity	Obesity
		Colon	Azelaic acid	ACIII/PKA/CREB	*In vivo*	GM modulation ↑GLP-1 secretion	Inflammatory bowel disease
		Liver	Eugenol	ACIII/PKA/CREB/HSL	*In vivo*	↑hepatic FAO; ↑lipolysis and GM modulation	Non-alcoholic fatty liver disease
OR6A2	Olfr2	Vascular	Octanal	ACIII/cAMP/CNG Ca+2 channels	*In vitro In vivo*	↑NLRP3 activation; ↑atherosclerosis; ↑AAA	Atherosclerosis
OR10J5	Olfr16	Liver	Alpha-cedrene	ACIII/PKA/CREB/HSL	*In vivo*	↓hepatic lipid content and ↑hepatic FAO	Obesity
OR2AT4	​	Skin	Sanddalwood	ACIII/cAMP/CNG Ca+2 channels	*In vitro*	↑wound-healing processes	Wound healing
OR2L13	Olfr168	Platelets	CCF0054500	cAMP	*In vitro*	↓thrombosis	CVD

The GM metabolites entering the bloodstream circulate in the body and reach the OE. Thus, with the nasal microbiome (NM) (Lazarini et al., 2022), they could act on OE structure similar to what is observed between GM and the intestinal epithelium (Wikoff et al., 2009; Davidson et al., 2017). NM could affect diverse steps of the odorant detection. Indeed, microorganisms present in OE mucus may i) modulate the OSN turn-over and consequently the OSN population properties, impacting on odorant detection, ii) change the odorant accessibility to the OSN cilia, iii) modify the rate of odorants degradation by metabolizing them or influencing the expression of the xenobiotic metabolizing enzymes, and iv) release odorants (Lee et al., 2011; Ezenwa and Williams, 2014; Casadei et al., 2019). The influence of microbiomes on olfaction has been investigated in germ-free mice. The authors observed that the OE thickness was unaltered, the cilia layer was thinner, the expression of genes involved in the olfactory transduction pathway was decreased, the OBP expression was higher, and the electro-olfactogram signal amplitude for diverse odorants was stronger than that in wild-type animals (François et al., 2016). Since the GM is the most representative component of the host microbiome, it can be speculated that these differences mainly arise from its absence in germ-free mice. Different external conditions (such as geography, air pollution, diet, and the history of tobacco use), antibiotic use and exposure to viruses and xenobiotics affect the NM composition, eventually allowing pathogen invasion in the brain (Lazarini et al., 2022; Kraemer et al., 2019). Therefore, the NM is now recognized as a patrol of brain health through olfactory neural pathways, systemic immune signaling, and inter-organ communication within the gut–lung–brain axis [for an in-depth discussion, refer to Thangaleela et al. (2022), Vásquez-Pérez et al. (2025), Mathias et al. (2026), and Mukhtar et al. (2026). Despite these data linking GM and NM diversity and metabolites, significant knowledge gaps remain, including i) the incomplete mechanistic validation in a translational model, ii) limited availability of longitudinal clinical studies able at establishing causality, and iii) deficient characterization of the real impact of environmental exposures and aging on the microbiome–nasale interface. Indeed, experimental confirmation of key pathways persists incomplete, specifically in experimental models that mirror human aging and disease.

Finally, Orecchioni et al. (2022) demonstrated that octanal, a medium-chain aliphatic aldehyde endogenously produced through lipid peroxidation during oxidative stress (Rizzo, 2014), is a ligand of OR6A2/Olfr2 detected in human and mouse vascular macrophages of atherosclerotic plaques, respectively. In mice, octanal treatment induced a significant increase in the atherosclerotic lesion area and in TNF and IL-1beta plasma concentrations, by activating the nucleotide-binding oligomerization domain (NOD)-like receptor (NLR) family pyrin domain containing 3 (NLRP3) inflammasome (Table 1). Conversely, genetic Olfr2 deletion in the bone marrow led to a reduction and stabilization of aortic plaques (Orecchioni et al., 2022). Furthermore, the authors found that in mice and humans, the levels of octanal were positively correlated with total cholesterol and triglyceride concentrations, suggesting that octanal could represent a new biomarker for atherosclerosis development and progression (Orecchioni et al., 2022). Similarly, a recent study showed that Olfr2 regulates monocyte recruitment and macrophage-driven inflammation during abdominal aortic aneurysms (AAAs), and the inhibition or activation of the receptor protects from or worsens AAA, respectively (Schelemei et al., 2026).

## 6 Tissue-specific signaling pathways

### 6.1 The nasal OR signaling pathway

**FIGURE 2:**
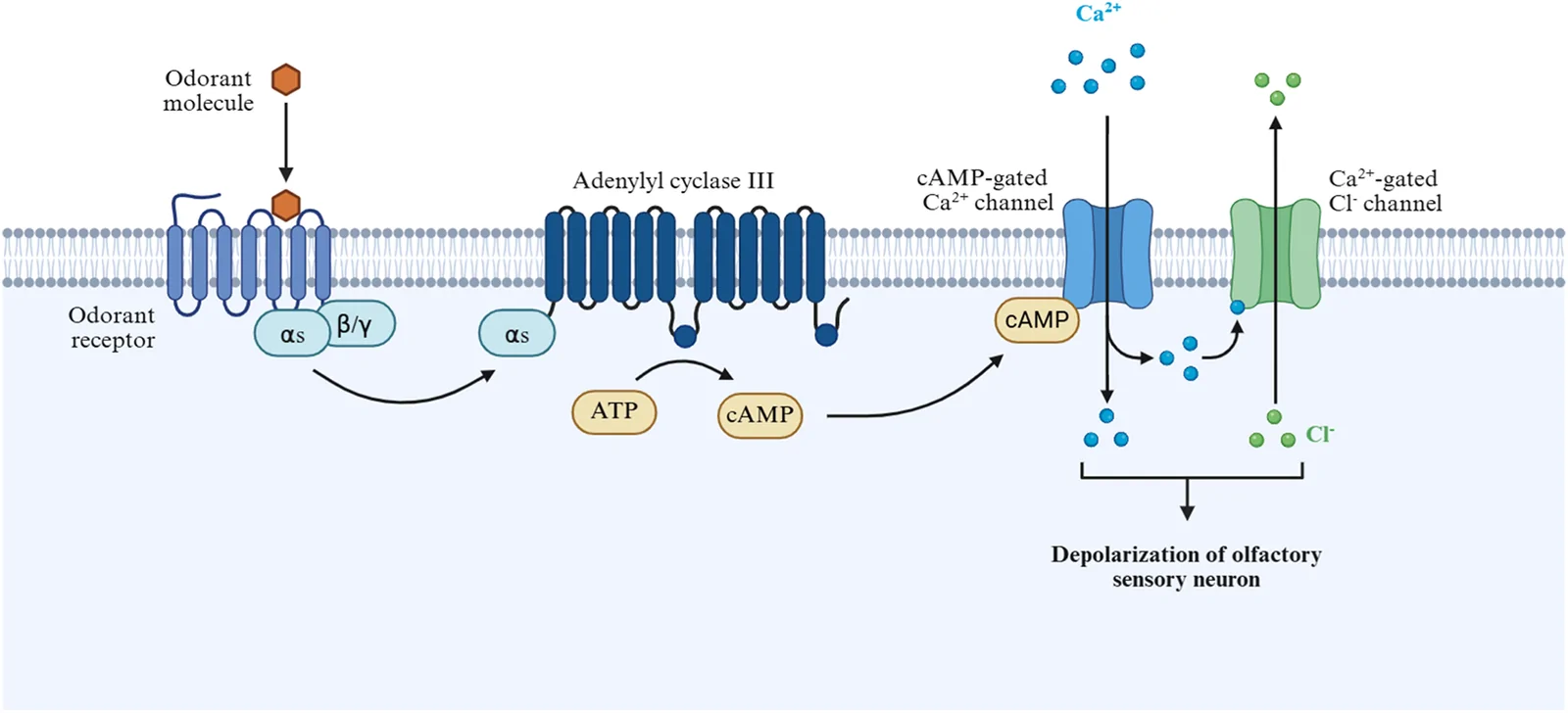
Sensory transduction in the nasal epithelium. Signaling pathway of ORs triggered by odorants in the main olfactory epithelium. Following the binding to its ligands, the OR promotes the activation of adenylyl cyclase type III that stimulates the synthesis of cAMP from ATP. This increase in the cAMP concentration opens the cyclic nucleotide-gated Ca^2+^ channel, leading to an increase in intracellular Ca^2+^ levels, further stimulating the opening of the calcium-activated chloride channel. Eventually, this exchange of ions causes depolarization of olfactory sensory neurons, the generation of action potential, and neuronal impulse transmission to the central nervous system. Please refer to the text for the meaning of all abbreviations used in the figure. Figure created with BioRender.com.

Each OSN expresses a single OR, which binds several odorants; conversely, odorants can bind to several ORs (Malnic et al., 1999). ORs are class A seven-transmembrane G protein-coupled receptors (GPCRs), and the mammalian genome contains as many as 1,000 genes, making them the largest family of GPCRs and probably the largest gene family in the entire genome (Buck and Axel, 1991). Studies showed that odorants bind to ORs mainly through hydrophobic and van der Waals interactions; because these interactions are relatively weak, they are consistent with the finding that odorant dwell times are extremely short (Katada et al., 2005; Bhandawat et al., 2005). The complex, OR/odorants, promotes the dissociation of the GTP–Galphas subunit from the alpha–beta–gamma heterotrimer and the activation of the adenylyl cyclase type III (ACIII) that stimulates the synthesis of cyclic adenosine monophosphate (cAMP) from adenosine triphosphate (ATP). This increase in cAMP levels triggers the Ca^2+^ influx through cyclic-nucleotide-gated (CNG) channels and the opening of Ca^2+^-activated Cl^−^ (CAC) channels, eventually leading to cell depolarization, generation of an action potential, and neuronal impulse transmission to the CNS (Figure 2) (Stephan et al., 2009; Reisert et al., 2005). Indeed, mutations in G proteins, ACIII, and CNG channels cause impairment of the olfactory function, providing evidence that the cAMP-dependent signaling pathway is the primary transduction mechanism in the OE (Touhara and Vosshall, 2009). Finally, Ca^2+^-mediated feedback inhibition of the CNG channel, activation of phosphodiesterase, and inhibition of the ACIII, together with extrusion of Ca+2, are the signal termination of the OR pathway (Bradley et al., 2005). In addition, the dissociated G beta–gamma heterodimer can bind to G-protein-coupled receptor kinases (GRKs), promoting the phosphorylation of serine and threonine residues localized within the third intracellular loop and the carboxyl-terminal tail domains of the receptor, followed by the binding of cytosolic cofactor proteins known as arrestins (Mashukova et al., 2006; Luttrell et al., 2001). This phosphorylation usually starts with the OR internalization, eventually leading to protein degradation (downregulation) or receptor recycling to the cell surface (re-sensitization) (Escribá et al., 2007).

### 6.2 Major signaling pathways regulated by ecnomotopic ORs

**FIGURE 3:**
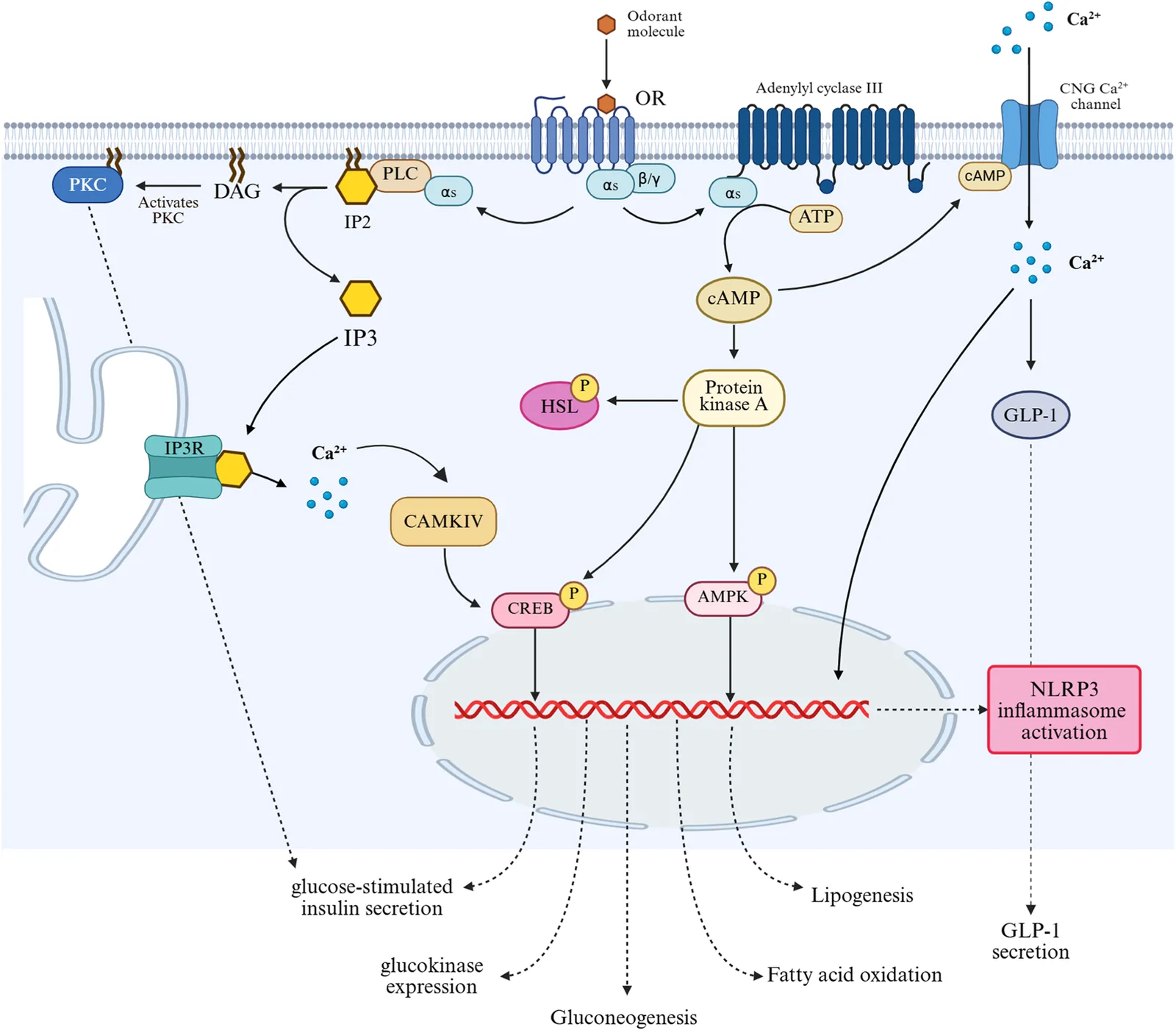
Signaling pathways of ecnomotopic ORs. The binding of ligands to ecnomotopic ORs is not associated with cell depolarization and neuronal pulse generation but linked to the activation of different signaling pathways, which depend on the cell types and the signaling components involved. In metabolic cells, such as hepatocytes, adipocytes, and pancreatic, enteroendocrine, and renal tubule cells, adenylyl cyclase type III/protein kinase A (ACIII/PKA) and phospholipase C/inositol 1,4,5-triphosphate (PLC/IP3) downstream signaling are the major pathways. Please refer to the text for the meaning of all abbreviations used in the figure. Figure created with BioRender.com.

The binding of ligands to ecnomotopic ORs is not associated with cell depolarization and neuronal pulse generation (described above) but linked to the activation of different signaling pathways, which depend on the cell types and the signaling components involved. In metabolic cells, such as hepatocytes, adipocytes, and pancreatic, enteroendocrine, and renal tubule cells, ACIII/protein kinase A (ACIII/PKA) and phospholipase C/inositol 1,4,5-triphosphate (PLC/IP3) downstream signaling are the major pathways involved (Figure 3) (Marinissen and Gutkind, 2001; Urbani et al., 2022). In ACIII/PKA, active ACIII produces cAMP, which promotes PKA activation (Sassone-Corsi, 2012), resulting in phosphorylation/activation of different cytosolic [hormone-sensitive lipase (HSL)] and nuclear [AMP-activated protein kinase (AMPK) and cAMP-response element binding protein (CREB)] proteins (Li et al., 2019; Wu et al., 2017; Tong et al., 2017; Tong et al., 2019; Wu et al., 2015; Wu et al., 2019). The cAMP may also stimulate the opening of CNG channels expressed on the endoplasmic reticulum, eventually increasing the intracellular Ca^2+^ levels, inducing GLP-1 secretion (Han et al., 2018) or NLRP3 inflammasome activation (Orecchioni et al., 2022). On the other hand, in the PLC/IP3 pathway, the GTP–Galphaq, dissociated from the alpha–beta–gamma heterotrimer, activates the phospholipase C (PLC), triggering the formation of 1,2-diacylglicerol (DAG) and IP3. DAG activates the protein kinase C (PKC) that upregulates the glucose-stimulated insulin secretion (GSIS), whereas IP3 binding to the IP3 receptor, expressed on the endoplasmic reticulum, promotes the calcium release, which activates Ca+2/calmodulin-dependent protein kinase IV (CAMKIV) that phosphorylates the CREB, eventually inducing glucokinase (GK) expression and increasing glucose absorption and GSIS (Figure 3) (Gong et al., 2025).

## 7 Olfactory impairment and neurodegenerative/metabolic syndromes

Healthy olfactory pathways need intact function and anatomy within the nose itself, the OE, the OB, and the central brain structures in charge of olfactory processing (Yoshikawa et al., 2018; Larsson et al., 2004). Age-related olfactory dysfunction, known as presbyosmia, is a common sensory impairment in aging adults. Moreover, evidence suggests that hyposmia/anosmia is a consequence of the odorant inability to reach the OSNs, as well as of direct damage to the olfactory mucosa, mainly the OSNs (Raviv and Kern, 2004). Environmental stimuli, aging, and cellular senescence contribute to the progress of a decreased response of olfactory structures. During the aging process, the OSN number decreases because the OE is gradually replaced by the respiratory epithelium, a phenomenon known as olfactory metaplasia (Paik et al., 1992; Fitzek et al., 2022). Experimental data suggest that this tissue modification mirrors what is observed in models of chronic olfactory mucosal inflammation and is triggered by inflammatory-mediated changes in olfactory stem cell progenitors (Lane et al., 2010; Oliva et al., 2022). Moreover, olfactory impairment has been detected in (and is one of the stronger predictors of) various neurodegenerative disorders (Dintica et al., 2019), as well as in diabetic and obese subjects (Faour et al., 2022). Although multiple factors contribute to this association, the shared hallmark of these conditions appears to be a chronic, sterile inflammatory microenvironment (Parolini and Amedei, 2026; Fava et al., 2025). In addition, there are extensive data showing that the relationship between mammalian olfaction and metabolism appears to be a bidirectional neurocircuit process, in which olfaction is enrolled in the control of food intake, energy expenditure, and fat metabolism, and, at the same time, olfactory discrimination is regulated by nutritional status and variations in the concentration of neuropeptides (such as leptin, insulin, and ghrelin) that modulate energy homeostasis (Jovanovic et al., 2022). Indeed, anorexigenic hormones (e.g., insulin and leptin) diminish odorant sensitivity (Lacroix et al., 2008; Savigner et al., 2009), which is, on the contrary, increased a few minutes after the application of orexigenic peptides, for instance, orexin and neuropeptide Y (NPY) (Pru et al., 2009; Negroni et al., 2012). However, even though insulin, ghrelin, leptin, orexin, and NPY receptors have been detected in the olfactory mucosa and OB (Pru et al., 2009; Aimé et al., 2012), the mechanisms regulating this interplay remain poorly understood.

Of note, olfactory loss influences nutrition, safety, and social relationships, and subjects with olfactory impairment have been shown to be at greater risk for overall mortality (Raviv and Kern, 2004). Different epidemiological and clinical factors have been associated with presbyosmia (Larsson et al., 2004; Karpa et al., 2010; Mobley et al., 2014). Van Regemorter et al. (2020) have recently reviewed the main hypothesis proposed to explain the age-related olfactory dysfunction. Among these, it is worth highlighting the role of the the apolipoprotein (apo) E4 allele, a well-known risk factor for AD development (Yoshikawa et al., 2018) and the presence of comorbidities (Van Regemorter et al., 2020). Indeed, the combination of the apoE4 allele and olfactory impairment in elderly persons without dementia strongly drives the decline in global cognitive function (Olofsson et al., 2009), and olfactory impairment has been detected in individuals with systemic and endocrine diseases (such as hypothyroidism and type 2 diabetes mellitus), chronic kidney diseases, or liver failure (Landis et al., 2004). Beyond clinical anosmia, modifications in the OR expression within the OE have been directly involved in neurodegenerative disorder pathology. Transcriptomic analyses detected a lower OR expression, along with an impairment of the canonical olfactory transduction in the olfactory mucosa in patients with AD and PD compared to that in healthy controls (Ferrer et al., 2016; Attems et al., 2005; Devanard et al., 2015). These data were corroborated by experimental studies using a conditional knockout model of AD and PD, which showed that loss of the GTP–Galphas subunit or ACIII accelerated synaptic dysfunction and neuroinflammation (Dubey et al., 2026). In addition, alpha-synuclein aggregates originate in specific regions, the enteric nervous system and the OB, and spread to connected brain areas *via* intra-axonal transport. Of note, prodromal PD symptoms, such as constipation and hyposmia, correspond to these sites of pathology (Poewe et al., 2017). Post-mortem studies validated neuronal loss in the OB and associated areas (Pearce et al., 1995; Harding et al., 2002; Silveira-Moriyama et al., 2009). Longitudinal studies suggest that hyposmic patients are at increased risk for motor complications, such as gait freezing, and emphasize the utility of serial olfactory testing, odorant identification, discrimination, and threshold to improve patient stratification at diagnosis (Lee et al., 2021; Vaswani et al., 2022). Studies in AD confirm these results. Indeed, a greater decline in olfaction is linked to a faster accumulation of amylod-beta and tau in areas related to olfaction, and a decrease in odor identification is directly related to altered cerebrospinal fluid biomarkers (Tian et al., 2022; Lafaille-Magnan et al., 2017). Different studies indicated that subjects with cognitive decline have a significant odor identification impairment compared to controls (Jobin et al., 2021; Sohrabi et al., 2009; Wang et al., 2021). One study reported that combining odor identification assessments with neuropsychological testing achieved 100% sensitivity and 80%–90% specificity in predicting AD (Lojkowska et al., 2011). However, further research is needed to understand the role of olfactory dysfunction in these neurodegenerative diseases, as well as in other common diseases, such as amyotrophic lateral sclerosis, multiple sclerosis, and Huntington’s disease, and to clarify the type of this association, which is causal, compensatory, or associative. Meanwhile, current olfactory testing procedures and functional magnetic resonance imaging protocols need to be further refined to provide a comprehensive assessment of olfactory dysfunction, including odor identification, discrimination, and detection thresholds, while reducing subjectivity through the integration of electrophysiological measures (Rombaux et al., 2012; Lee et al., 2020). These improvements may help define the optimal combination of olfactory, cognitive, and imaging biomarkers required to advance early screening and therapeutic strategies.

Loss-of-function experiments also confirm that ecnomotopic ORs are functionally integrated into tissue-specific signaling pathways. For instance, Olfr16 deficiency exacerbates hepatic steatosis in mice, specifically under a high-fat diet (Parolini et al., 2014), by inhibiting the PKA/AMPK/CREB/HL pathways and upregulating the genes involved in lipogenesis and fatty acid uptake (Kang et al., 2024). Conversely, Olfr16 activation by alpha-cedrene reduces hepatic lipid concentration through PKA/AMPK/CREB/HL activation (Table 1) (Tong et al., 2017; Tong et al., 2019). In addition, the knockdown of OR10J5 (human ortholog of Olfr16) in human hepatocytes caused an increase in lipid accumulation (Tong et al., 2017). Griffin et al. (2009) documented that Olfr16 is a key regulator of myogenesis through its action on cell migration and adhesion. In addition, Olfr16 appears to be needed for proper skeletal muscle regeneration as Olfr16 deletion causes myofiber branching, commonly associated with muscular dystrophy (Griffin et al., 2009). Busse et al. (2014) demonstrated that sandalone, a synthetic sandalwood odorant, is an OR2AT4 agonist, and its stimulation triggers Ca^2+^ influx and wound-healing processes in human keratinocytes (Table 1). Conversely, OR2AT4 silencing in the same human cells abolishes the effects of sandalone on cell proliferation, migration, and regeneration (Busse et al., 2014).

In summary, these results emphasize that OR expression changes in the OE and the major organ systems are not simply correlated to smell loss but may be actively involved in disease development and progression by altering sensory input, systemic inflammation, and circuit vulnerability. Integrating olfactory function screening, such as odor identification, olfactory threshold, and odor discrimination, and OR datasets into multi-omic frameworks will allow the clinicians to include the olfactory biology in updated diagnostic guidelines, leading to a better stratification of patients and eventually individualized therapeutic protocols.

## 8 Novel therapeutic strategies targeting the OR pathways

**FIGURE 4:**
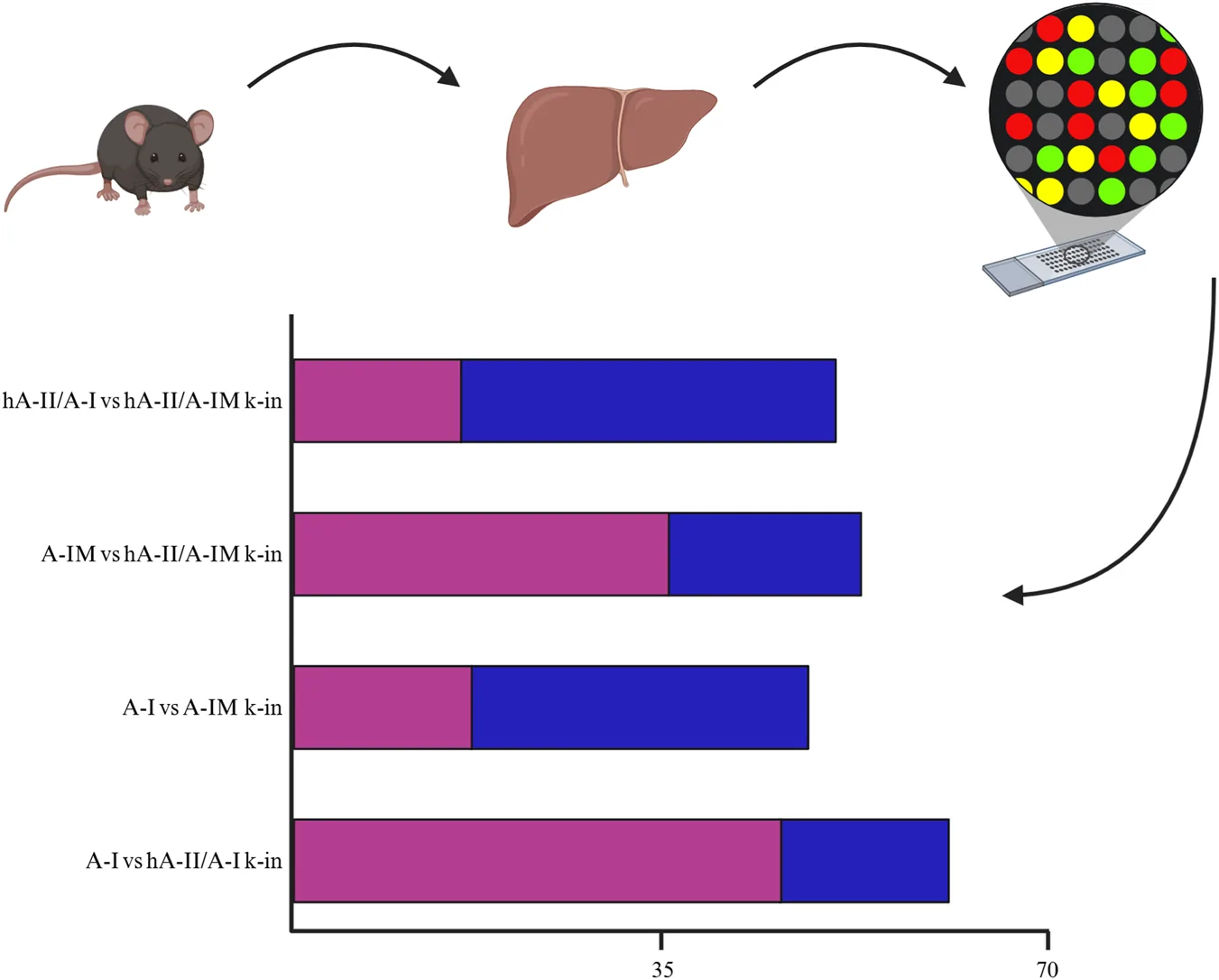
Hepatic differentially expressed genes (DEGs). Numbers of upregulated (blue) and downregulated (pink) DE olfactory receptor (Olfr) genes in the mouse livers of hA-II/A-I k-in *versus* hA-II/A-IM k-in, A-I *versus* hA-II/A-IM k-in, A-I k-in *versus* A-IM k-in, and A-I k-in *versus* hA-II/A-I k-in mice. The horizontal axis represents the number of genes. Figure created with BioRender.com.

By using transcriptomic analyses and genetically modified mouse models, two independent studies found that the liver and atherosclerotic lesion macrophages express the same Olfrs, in terms of number and types (Amedei and Parolini, 2026; McArdle et al., 2019). Interestingly, the first study documented for the first time that the expression of hepatic Olfrs and the genes involved in the downstream Olfr pathways was influenced by human apolipoproteins (Amedei and Parolini, 2026). In detail, human apoA-II (hAII) and apoAIMilano (AIM), the first molecular variant of human apoAI (AI) (Parolini et al., 2012; Ganzetti and Parolini, 2023), significantly up- and downregulated the hepatic gene expression of Olfrs, by acting as “transcription factors” and/or “mRNA modulator (Figure 4) (Amedei and Parolini, 2026). Among the upregulated genes, the authors found that none of them have been characterized, whereas among the downregulated genes, four Olfrs were previously discovered and/or characterized, namely, Olfr43 (Wu et al., 2017; Wu et al., 2021), Olfr99, Olfr1366, and Olfr646 (Amedei and Parolini, 2026). Of note, Zhao et al. (2023) showed that the hepatic Olfr646 expression was significantly modified by inulin, the most common prebiotic which can modulate the GM milieu through the SCFA production (Illiano et al., 2020). Keeping in mind that these apolipoproteins are the major determinant of the biological activities of high-density lipoprotein (HDL) and that the most prescribed cholesterol lowering agents, namely, statins (Marchesi et al., 2011; Parolini, 2025b), increase HDL levels, these results may shed new light on and open new frontiers in cardiovascular disease (CVD) prevention and update CVD clinical guidelines.

Similarly, the second study (McArdle et al., 2019) found that the Olf2 gene expression and that of its downstream signaling molecules were significantly increased after treatment with lipopolysaccharides (LPSs). The authors speculated that LPS sensing through toll-like receptor 4 (TLR4) promotes the activation and nuclear translocation of NF-kB and the AP-1 complex, eventually leading to the upregulation of Olfr2 expression, along with that of pro-inflammatory cytokines and antimicrobial peptides (McArdle et al., 2019). These results helped, at least in part, clarify the pro-atherogenic role exerted by Olfr2 (Orecchioni et al., 2022). Therefore, OR6A2, the human ortholog of Olfr2, could serve as a new therapeutic target for decreasing inflammation triggered by lipids for the atherosclerosis prevention and treatment (Orecchioni et al., 2022). Furthermore, it is known that cardiovascular-related deaths continue to increase due to the residual cardiovascular risk, driven essentially by the inflammation (Orecchioni et al., 2022) and the high residual platelet reactivity (HRPR), this latter being a risk for thrombosis (Rana et al., 2019). Aggarwal et al. (2026) have recently identified a specific agonist of OR2L13, an OR expressed in adult platelets that is stored in alpha granules before membrane surface translocation upon disturbed blood flow (Morrell et al., 2022). This study demonstrated that this OR2L13 ligand inhibits thrombosis without impairing hemostasis, by depolymerizing the platelet actin cytoskeleton to limit platelet shape change, in isolated platelets, platelet-rich plasma, and whole blood from both humans and mice (Table 1). In addition, in a murine model of myocardial infarction, the authors confirmed the presence of persistent HRPR associated with increased platelet-surface Olfr168 (murine OR2L13 ortholog) expression, which was suppressed by the ligand administration. Finally, this effect was not observed in mice lacking *Olfr168*, further corroborating the specificity of this new molecule (Aggarwal et al., 2026). In other words, this evidence suggests that the development of a platelet OR2L13-modulating ligand may represent a new alternative for antiplatelet therapy in patients who have a higher HRPR incidence when using standard-of-care treatments.

Altogether, these (*in vitro* and *in vivo*) studies highlighted the feasibility of modulating physiological pathways throughout OR activation. Therefore, it is plausible to speculate that these effects could be translated to humans. However, different factors should be considered by researchers before developing a clinical protocol. First, all the *in vitro* data must be confirmed in diverse experimental models. Second, additional *in vivo* experiments should be performed to better reproduce the human conditions, aiming at finding the therapeutic range for dose administration and treatment time.

## 9 Future perspectives and translational limitations

Deorphanizing the human ORs and unveiling their downstream signaling pathways are the major gaps in OR research. Dubey et al. (2026) and Gu et al. (2025) described that traditional approaches, such as heterologous expression and reporter-based assays, should be integrated with AI and ML frameworks to systematically combine receptor genotypes, ligands, and tissue-specific functions. The next step will be the integration of AI/ML ligand-prediction pipelines with spatial transcriptomics (ST), aiming to strengthen confidence in the predictions by confirming that the receptor is expressed in relevant tissue or disease-affected sites. Indeed, ST through *in situ* receptor expression allows the localization of both nasal and ecnomotopic ORs within intact anatomical and cellular architecture. In parallel, cryo-EM, a high-resolution imaging technique able to identify the near-atomic structures of biological macromolecules is critical for unveiling how the odorants can interact and activate the ORs, providing data for homology modeling and training of AI-based prediction algorithms (Dubey et al., 2026; Gu et al., 2025). Following this path, in the past 3 years, significant advances have been made in the structural OR analysis. For example, both the structures of the active (complexed with propionate) and inactive human OR51E2 have been elucidated using cryo-EM and structural predictions generated by ML algorithms, respectively (Billesbølle et al., 2023; Alfonso-Prieto and Capelli, 2023). Furthermore, De March et al. (2024) determined the structure of the consensus OR51cs (engineered OR crafted) by using a consensus protein design strategy, whereas the structure of the consensus OR52cs has been determined using cryo-EM, in both ligand-free and octanoate-bound states (Choi et al., 2023). A 3D transcriptomic atlas of the mouse olfactory mucosa has been obtained, allowing to establish that Olfr genes are spatially distributed in continuous overlapping zones and that the spatial distribution correlates with odorant mucus solubility (Dubey et al., 2026). Finally, a two-stage structure-based virtual screening approach has been used to predict novel ligands for OR51E2 (Jabeen et al., 2020), whereas a sensitive split luciferase assay has been used to monitor OR responses to ligands, independent of the conventional cAMP production pathways (Tanazawa et al., 2025). These studies proved that this “ensemble” could speed the process to fully understand the relationship between tissue expression, type of receptor, and downstream signaling pathways under physiological and pathological conditions, eventually leading to the design of novel therapeutic targets for treating neurodegenerative and metabolic disorders.

Despite these advancements, structural and functional studies of ORs are still challenging due to the limited information that is seminal to serve as crucial reference and support for homology modeling and training of AI-based algorithms. This could lead to the misinterpretation and/or technical-based bias of the OR–ligand pairs, their activations, and downstream physiological or pharmacological outcomes. Therefore, we urgently need to confirm and validate these new data under different experimental conditions to fill this gap.

## 10 Conclusion

In the olfactory system, molecular detection is the primary occupation and is most highly developed. Evidence suggests that the olfactory mucosa must be recognized as a crucial player of pathogen defense, being a component of the upper respiratory tract, as well as a mucosal barrier. Immune protection of the olfactory mucosa is crucial for addressing respiratory pathogen transmission and neurotrophic microbial invasion. A deeper understanding of the olfactory mechanisms will allow us to redesign ORs as clinically actionable molecular systems rather than peripheral sensory curiosities. Their great polymorphism, ligand-driven adaptability, and ecnomotopic expression in different tissues place ORs as key mediators bridging environmental exposures to metabolic, immunological, neurological, and oncological clinical manifestations. High-throughput functional technologies, AI-enabled receptor and ligand modeling, and engineered biosensing platforms prove that OR biology should be recognized as a new marker to be included into precision medicine strategies. However, several gaps still need to be filled, ranging from incomplete ligand deorphanization to interpretability of chemosensory-linked data. Of course, AI approaches are suitable for overcoming some technical limitations, even though ensuring privacy and equity are features that need to be addressed. Furthermore, the data from large-scale longitudinal trials are critical to link sensory genotypes to healthy paths and to corroborate ORs as new biomarkers worthy of being included in up-to-date therapeutic guidelines. Finally, recent data highlighting the feasibility of OR expression modulation should be implemented in different experimental models and validated in well-designed and tailored clinical trials with long-term follow up, to rightly open a new epoch in patient-centered care.

In summary, these data show that a holistic vision should be taken up considering that the multi-omics advances enable integrative analyses from various biological sources, aiming to identify a personalized molecular signature.
